# The Prognosis Analysis of Liver Cirrhosis with Acute Variceal Bleeding and Validation of Current Prognostic Models: A Large Scale Retrospective Cohort Study

**DOI:** 10.1155/2020/7372868

**Published:** 2020-08-16

**Authors:** Yan Zhao, Mudan Ren, Guifang Lu, Xinlan Lu, Yan Yin, Dan Zhang, Xin Wang, Wenhui Ma, Yarui Li, Guohong Cai, Yiguang Lin, Shuixiang He

**Affiliations:** ^1^Department of Gastroenterology, First Affiliated Hospital of Xi'an Jiaotong University, Xi'an, China; ^2^Department of Neurobiology, School of Basic Medicine, Fourth Military Medical University, Xi'an, China; ^3^School of Life Sciences, University of Technology Sydney, Australia

## Abstract

**Background:**

Acute variceal bleeding is a major cause of death in liver cirrhosis. This large scale retrospective cohort study aims to analyze the prognosis of patients with cirrhosis and acute variceal bleeding and to validate the current prognostic models.

**Methods:**

Patients with cirrhosis and acute variceal bleeding were enrolled from Jan 2019 to March 2020. The independent prognostic factors for in-hospital death were identified by logistic regression analyses. Area under curves (AUCs) was compared among Child-Pugh, cirrhosis acute gastrointestinal bleeding (CAGIB) score, and model for end-stage liver disease (MELD) and neutrophil-lymphocyte ratio (NLR) scores.

**Results:**

Overall, 379 patients with liver cirrhosis and acute variceal bleeding were consecutively evaluated. The majority of the patients were males (59.1%) and the mean age of all patients were 53.7 ± 1.3 years (range 14-89). Hepatitis B virus (HBV) was the most common underlying cause of liver cirrhosis (54.1%). 72 (19%) patients had hepatocellular carcinoma. Multivariate logistic regression analyses showed that age, HCC, WBC, total serum bilirubin, serum creatinine, and ALT were independently associated with in-hospital death. And the odds ratios (ORs) for in-hospital death were 1.066 (95% CI 1.017-1.118, *P* = 0.008), 7.19 (95% CI 2.077-24.893, *P* = 0.001), 1.123 (95% CI 1.051-1.201, *P* = 0.001), 1.014 (95% CI 1.005-1.023, *P* = 0.003), 1.012 (95% CI 1.004-1.021, *P* = 0.006), and 1.005 (95% CI 1.000-1.009, *P* = 0.036), respectively. In the whole cohort with HCC patients, the AUCs of Child-Pugh, CAGIB, MELD and NLR scores were 0.842 (95% CI 0.801-0.878), 0.840 (95% CI 0.799-0.876), 0.798 (95% CI 0.754-0.838), and 0.688 (95% CI 0.639-0.735), respectively. The differences were statistically significant between Child-Pugh and NLR scores (*P* = 0.0118), and between CAGIB and NLR scores (*P* = 0.0354).

**Conclusion:**

Child-Pugh and CAGIB scores showed better predictive performance for prognosis of patients with cirrhosis and acute variceal bleeding than NLR scores.

## 1. Introduction

Acute variceal bleeding is a frequent medical emergency with the 6-week mortality of 15-20% in patients with liver cirrhosis [1, 2]. Hepatocellular carcinoma (HCC) is one of the most common tumors worldwide with approximately 850,000 new cases each year [3]. The HCC patients with cirrhosis may suffer from both the tumor burden and variceal bleeding associated with liver cirrhosis. Combined treatment with prophylactic antibiotics, vasoactive drugs, endoscopic techniques, and interventional treatments are the recommended therapy methods for patients with acute variceal bleeding. However, treatment failure remains as high as 20% [4].

The consensus suggested the importance of early use of risk stratification scores in patients with acute upper gastrointestinal bleeding, which could help reduce the costs and resources without influencing the outcomes of patients [5]. Conventional scoring systems with acute upper gastrointestinal bleeding included Glasgow-Blatchford score (GBS), Rockall score, and AIMS65 score [6–8]. However, these systems were not designed for patients with cirrhosis. As we know, variceal bleeding is the most frequent reasons of acute upper gastrointestinal bleeding in patients with liver cirrhosis. Recent studies have shown that these scoring systems were successful for predicting mortality risk in patients with nonvariceal upper gastrointestinal bleedings [9, 10]. Oakland et al. developed a new scoring system based on the data from Canada, the United Kingdom, and Australia (CANUKA), which was used to identify low-risk patients with 30-day rebleeding or death [11]. Tammaro et al. developed T-score to predict high-risk endoscopic stigmata and the need for early intervention [12]. Robertson et al. validated the AIMS65 score and found that AMIS65 score was equivalent to other liver disease severity risk stratification scores in predicting short term mortality [13]. However, these scoring systems were designed for acute upper gastrointestinal bleeding rather than for liver cirrhosis patients with acute variceal bleeding. Although multiple scoring systems have been proposed about liver diseases or acute upper gastrointestinal bleeding, very limited data are available for the prognostic value of current scoring systems in patients with acute variceal bleeding.

Child-Pugh, model for end-stage liver disease (MELD), and neutrophil-lymphocyte ratio (NLR) scores have been widely used in clinical practice considering they were used for prognostic assessment in patients with liver cirrhosis. Child-Pugh score was proposed to predict the risk of surgery for patients with variceal bleeding. MELD score was designed to predict the prognosis of patients who received transjugular intrahepatic portosystemic shunts (TIPS) therapy. Currently, it has been widely used to rank the priority of liver transplantation candidates. NLR is a scoring system through evaluating the degree of inflammation reaction and has been considered as a marker for the severity of liver fibrosis and cirrhosis. Lately, cirrhosis acute gastrointestinal bleeding (CAGIB) was proposed by Bai et al. They use a large scale of patients with cirrhosis and acute gastrointestinal bleeding to propose and validate the performance of CAGIB score. And their results showed that CAGIB score performed better than Child-Pugh, MELD, and NLR [14]. Although several previous studies have compared the discriminative abilities of the staging systems, it still remains controversial which could reflect the prognosis more accurately.

Therefore, we conducted this large cohort retrospective study to evaluate the prognostic factors for the liver cirrhotic patients with acute variceal bleeding and further to compare the discriminate ability of these current stage systems.

## 2. Methods

We screened all consecutive patients with acute gastrointestinal bleeding who were admitted to our hospital between January 2019 and March 2020. The inclusion criteria were acute variceal bleeding because of liver cirrhosis. The time frame for the acute bleeding episode should be 120 h (5 days) according to the Baveno V criteria [15]. The exclusion criteria were ulcer diseases, acute gastric mucosa hemorrhage, Mallory-Weiss syndrome, tumor diseases related bleeding, inflammatory bowel diseases, obscure gastrointestinal bleeding, and other reasons-caused bleeding. All consecutive patients who met these criteria were included. Because of the nature of this study, the informed written consent was waived. The following data were collected: age, gender, etiology, *α*-fetoprotein (AFP), history of GIB, hepatic encephalopathy (HE), ascites, and the laboratory tests at admission including white blood cell (WBC), platelet (PLT), hemoglobin (Hb), red blood cell (RBC), total bilirubin (TBIL), albumin (ALB), alanine aminotransferase (ALT), aspartate aminotransferase (AST), alkaline phosphatase (ALP), gamma-glutamyl transpeptidase (*γ*-GGT), prothrombin time (PT), international normalized ratio (INR), serum creatinine (Scr), and in-hospital death. Child-Pugh, CAGIB, MELD, and NLR scores were calculated for every patient, respectively [16–18].

MELD = 0.957 × log_e_ (creatinine mg/dL) + 0.378 × log_e_ (bilirubin mg/dL) + 1.120 × log_e_ (INR) + 0.643 × (cause of cirrhosis). For cause of cirrhosis, use 0 for alcohol-related liver disease or for cholestatic liver disease; 1 for all other causes.

(1) $$\begin{array}{c} \text{CAGIB}=\text{Diabetes }\left(\text{yes}=1,\text{no}=0\right)\times 1.040+\text{HCC }\left(\text{yes}=1,\text{no}=0\right)\times 0.974+\text{TBIL}\left(\mu \text{mol}/\text{L}\right)\times 0.005-\text{ALB }\left(\text{g}/\text{L}\right)\times 0.091+\text{ALT}\left(\text{U}/\text{L}\right)\times 0.001+\text{Scr }\left(\mu \text{mol}/\text{L}\right)\times 0.012-3.964. \end{array}$$

The NLR was calculated by dividing the absolute neutrophil count by the absolute lymphocyte count. NLR ≥5 was considered raised [19].

The Child-Pugh scores were consisted of encephalopathy, ascites, bilirubin, albumin, prothrombin time, and INR. The patients whose score 5 or 6 were good operative risks (grade A); 7, 8, or 9 moderate (grade B); and patients with 10-15 poor operative risks (grade C) [20].

### 2.1. Statistical Analysis

Continuous variables were summarized as the means and standard deviation. Categorical variables were expressed as frequencies and percentages. Logistic regression analyses were used to assess the prognostic values of the variables associated with in-hospital death. Odds ratios (ORs) with 95% confidence intervals (CIs) were calculated. Then, receiver operating characteristic curve (ROC) analysis was performed to evaluate the predictive performance of Child-Pugh score, CAGIB score, MELD score, and NLR score. The area under curve (AUC) was calculated. The predictive performance of each scoring system was compared. All statistical analyses were performed using SPSS software version 17.0 (IBM Corp, Armonk, NY, USA) and MedCalc software version 19.0.4 (MedCalc Software, Mariakerke, Belgium). *P* < 0.05 was considered statistically significant.

## 3. Results

### 3.1. Patients

**Figure 1:**
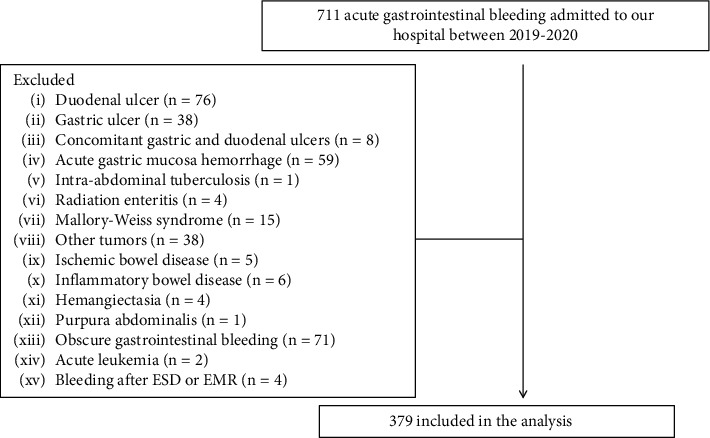
Patients flow chart.

Overall, we followed 711 consecutive patients with acute upper gastrointestinal bleeding who admitted to our hospital from Jan 2019 to March 2020. Of these patients, 379 with liver cirrhosis and acute variceal bleeding were consecutively evaluated (Figure 1). Detailed baseline clinical characteristics of these enrolled patients were provided in Table 1. The majority of the patients were males (59.1%). The mean age of all patients was 53.7 ± 1.3 years (range 14-89). Hepatitis B virus (HBV) was the most common underlying cause of liver cirrhosis (54.1%). 72 (19%) patients had hepatocellular carcinoma. Nine patients had undergone liver transplantation. 96 (25.3%) underwent TIPS treatment. 157 (41.4%) patients received endoscopic variceal ligation treatment, and 144 (38%) patients received gastric variceal obturation treatment.

### 3.2. Univariate and Multivariate Analyses

Univariate logistic regression analyses demonstrated that age, hepatitis infection, HCC, WBC, RBC, albumin, total serum bilirubin, serum creatinine, ALT, AST, alkaline phosphatase, Child-Pugh score, MELD score, NLR score, and CAGIB score were significantly associated with in-hospital death. Multivariate logistic regression analyses showed that age, HCC, WBC, total serum bilirubin, serum creatinine, and ALT were independently associated with in-hospital death. And the odds ratios (ORs) for in-hospital death were 1.066 (95% CI 1.017-1.118, *P* = 0.008), 7.19 (95% CI 2.077-24.893, *P* = 0.001), 1.123 (95% CI 1.051-1.201, *P* = 0.001), 1.014 (95% CI 1.005-1.023, *P* = 0.003), 1.012 (95% CI 1.004-1.021, *P* = 0.006), and 1.005 (95% CI 1.000-1.009, *P* = 0.036), respectively (Table 2). Child-Pugh score, MELD score, and NLR score are complex variables composed of many clinically significant variables, and therefore, they were not included in the multivariate analysis.

**Figure 2:**
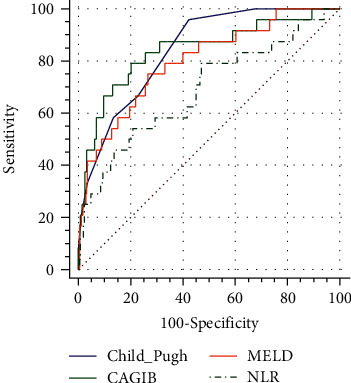
Comparisons of predictive performance of CAGIB score with Child–Pugh, MELD, and NLR score in liver cirrhotic patients including HCC. Blue line refers to the Child–Pugh score, green line refers to the CAGIB score, orange line refers to the MELD score, and black dotted line refers to the NLR score.

In the whole cohort including HCC patients, the AUCs of Child-Pugh, CAGIB, MELD, and NLR scores were 0.842 (95% CI 0.801-0.878), 0.840 (95% CI 0.799-0.876), 0.798 (95% CI 0.754-0.838), and 0.688 (95% CI 0.639-0.735), respectively (Figure 2). The differences were statistically significant between Child-Pugh and NLR scores (*P* = 0.0118), and between CAGIB and NLR scores (*P* = 0.0354).

**Figure 3:**
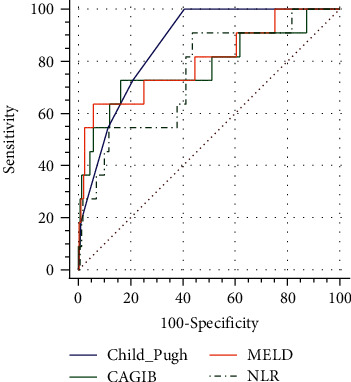
Comparisons of predictive performance of CAGIB score with Child–Pugh, MELD, and NLR score in liver cirrhotic patients without HCC. Green line refers to the Child–Pugh score, blue line refers to the CAGIB score, orange line refers to the MELD score, and black dotted line refers to the NLR score.

In the cohort without HCC patients, the AUCs of Child-Pugh, CAGIB, MELD, and NLR scores were 0.864 (95% CI 0.820-0.900), 0.780 (95% CI 0.729-0.826), 0.800 (95% CI 0.750-0.844), and 0.747 (95% CI 0.694-0.795), respectively (Figure 3). The differences between CAGIB, Child-Pugh, MELD, and NLR scores were not statistically significant.

## 4. Discussion

Acute variceal bleeding is a lethal complication of liver cirrhosis. Although some scoring models were used to predict the prognosis and mortality in liver cirrhosis and acute upper gastrointestinal bleeding, the prognostic scoring system for the mortality of patients with acute variceal bleeding was relatively rarely. The present work evaluated the prognosis of patients with liver cirrhosis and acute variceal bleeding and further validated the prognostic ability of current models. The strengths of this study were as follows: (1) the data was obtained from the large sample size and the patients with cirrhotic liver and acute variceal bleeding were consecutively enrolled; (2) we evaluated the prognosis of HCC patients with acute variceal bleeding; (3) most of the cases in our cohort were caused by HBV infection which differed from the patients in western countries; (4) this is the first study as an external validation of CAGIB score in patients with cirrhosis and acute variceal bleeding.

Currently, Child-Pugh, MELD, and NLR scores are the most widely known staging scores. Firstly, Child-Pugh is one of the oldest and useful tools utilized in clinical practice to estimate the prognosis of liver cirrhosis. Although Child-Pugh score has some limitations considering that it includes some subjective factors, such as ascites and hepatic encephalopathy which would be affected by therapy, it is still the most widely used prognostic scoring system for liver cirrhosis patients worldwide [21]. In our study, Child-Pugh was shown to be a reliable scoring system with the highest AUCs, which was higher than MELD, CAGIB, and NLR.

Secondly, considering all the patients with score more than 10 were classified as Class C in Child-Pugh system, it was suggested that Child-Pugh classification could not discriminate the patients with serious damaged liver function [17]. Under this background, MELD was created to predict the survival of patients after TIPS treatment and now has been used to evaluate the priority of liver transplantation [17]. A previous study by Salerno et al. demonstrated that MELD score performed better than Child-Pugh model in predicting short-term (3 months) outcome [22]. Then, the study by Schepke et al. suggested that there was only a slight difference in the predictive accuracy of 1-year survival between these two models [23]. However, some studies have shown that MELD correlated well with Child-Pugh score [24]. Moreover, the study by Serste et al. demonstrated that MELD score failed to predict the mortality in patients with refractory ascites [25]. The limitation of MELD is that it originated from advanced liver disease. And the calculation of MELD score is more complex compared with others. Thus, it still remains controversial about the advantage of MELD in clinical practice.

Thirdly, NLR is a scoring system that reflects the degree of inflammatory reaction with integrating two immune pathways. On one hand, neutrophils indicate the continuous inflammation; on the other hand, lymphocytes indicate the regulatory pathway [19]. NLR has been considered as a prognostic marker for patients with various tumors including HCC, gastric cancer, and lung cancer [26, 27]. The advantage of NLR is that the value of neutrophils and lymphocytes could be easily obtained in clinical practice. And as we know, the inflammatory reaction process plays an important role in the progression of liver fibrosis and cirrhosis. Thus, it was suggested that NLR could be used as a marker for the severity of liver fibrosis and cirrhosis. The systematic review by Peng et al. pointed that NLR was particularly associated with the degree of liver fibrosis in patients with nonalcoholic fatty liver disease (NAFLD) [28]. This is associated with the fact that inflammatory reaction is evolved in the progression of NAFLD. However, NLR failed to reflect other factors that may reflect the severity of liver damages.

CAGIB was recently proposed to predict the prognosis of patients with cirrhosis and acute gastrointestinal bleeding. It includes TBIL, ALB, Scr, ALT, diabetes, and HCC as variables predicting prognosis. In our study, we identified age, HCC, WBC, TBIL, Scr, and ALT as prognostic factors, which was similar with CAGIB score. In real-world practice, the rapid increase in Scr level indicated decreased kidney function. The importance of Scr level as a critical prognostic factor for patients with liver disease has been proved in previous studies [29, 30]. It was suggested that patients with renal failure and liver cirrhosis would had worse prognosis compared to patients with similar severity of liver disease [31]. In addition, in the training cohort and internal validation cohort in CAGIB score, there were around 14%-18% HCC patients, which was similar with the percentage of HCC patients in our cohort [14]. And HCC with over a 7-fold increased risk of in-hospital death played a crucial role in the prognosis.

All in all, a previous systematic review by Peng et al. compared the Child-Pugh and MELD scores in the evaluation of prognosis in patients with liver cirrhosis and found that both of them had similar prognostic value. However, these two scoring systems performed differently depending on specific conditions. They pointed that studies should illustrate clearly the candidates who should use Child-Pugh or MELD [16]. Our study showed that in the whole cohort including HCC, the AUCs of Child-Pugh and CAGIB were higher than that of MELD and NLR scores. And the differences reached statistically significant between Child-Pugh and NLR scores, and between CAGIB and NLR scores. These results implied that Child-Pugh and CAGIB had better performance than NLR in the evaluation of prognosis for patients with cirrhosis and acute variceal bleeding. We considered that the possible reason was the etiology of our patient mainly consisted of hepatitis infection rather than NAFLD. And 19% patients had tumor burden which was an important prognostic factor. NLR, as an index including inflammation markers, could not reflect accurately both the degree of liver function damage and the effect of tumor burden.

There are a few limitations to our study that need to be acknowledged. Firstly, this is a single-center study; the lack of data from multicenter may cause potential bias. Secondly, this is a retrospective study and patients received different treatments to stop bleeding. 25.3% patients received TIPS treatment, 41.4% patients received endoscopic variceal ligation treatments, and 38% patients received gastric variceal obturation treatments. The various treatment methods may possibly affect the prognostic. However, there was no treatment-related death in this study.

## 5. Conclusions

In conclusion, our study demonstrated that in the current models, Child-Pugh, CAGIB, and MELD had good prognostic ability in predicting the prognosis of liver cirrhotic patients with acute variceal bleeding. Child-Pugh and CAGIB performed better than NLR in the cohort including HCC patients.

## Figures and Tables

**Table 1:** Demographic, clinical, and biochemical characteristics in patients with acute gastrointestinal bleeding (*n* = 379).

	N
Age (*y*) (mean ± SD)	53.7 ± 1.3 (range 14-89)
Sex (male)	224 (59.1%)
Cause of cirrhosis	
Hepatic B virus	205 (54.1%)
Hepatic C virus	30 (7.9%)
Both hepatic B and C virus	2 (0.5%)
Autoimmune liver disease	42 (11.1%)
Alcoholic	9 (2.4%)
Other	91 (24%)
History of GIB	86 (22.7%)
Ascites	229 (60.4%)
Hepatic encephalopathy	9 (2.4%)
Hepatocellular carcinoma	72 (19%)
Baseline laboratory values, mean ± SD (range)	
White blood cell (×10^9^/L)	5.3 ± 5.3 (0.93-64.86)
Platelet (×10^9^/L)	78.2 ± 5.8 (1-511)
Red blood cell (×10^9^/L)	2.8 ± 0.7 (1.2-5.4)
Hemoglobin (g/L)	82 ± 2.4 (23-105)
Albumin (g/dL)	31.2 ± 5.9 (14.5-55)
Total serum bilirubin (*μ*mol/L)	36.7 ± 5.3 (2.3-662.4)
Serum creatinine (*μ*mol/L)	63.3 ± 4.3 (11-513)
International normalized ratio	2.2 ± 1.1 (0.97-1.74)
Alanine aminotransferase (U/L)	39.1 ± 6.5 (2.4-734)
Aspartate aminotransferase (U/L)	57.2 ± 1.2 (8-1244)
Alkaline phosphatase (U/L)	110.4 ± 1.1 (10-1425)
Gamma-glutamyl transpeptidase (U/L)	76.8 ± 2.1 (4.4-3331)
Prothrombin time (s)	17.2 ± 4.6 (1.2-80)
*α*-Fetoprotein (ng/mL)	1324.6 ± 1677.3 (0.19-287000)
Absolute neutrophil count (×10^9^/L)	4.2 ± 5.5 (0.5-58.8)
Absolute lymphocyte count (×10^9^/L)	4.2 ± 0.8 (-10.4)
Child-Pugh	
A	112 (29.6%)
B	205 (54.1%)
C	62 (16.4%)
Child-Pugh score	7.6 ± 1.7 (5-13)
MELD score	6.5 ± 0.7 (5.06-11.9)
NLR score	6.9 ± 9.3 (0.3-101.4)
CAGIB score	−5.6 ± 1.1 (-7.8-1.6)
In-hospital death	25 (6.6%)

MELD: model for end-stage liver disease; NLR: neutrophil to lymphocyte ratio; CAGIB: cirrhosis acute gastrointestinal bleeding.

**Table 2:** Predictors for overall survival in 379 patients with liver cirrhosis and GIB.

Variable	Univariate analysis			Multivariate analysis		
	OR	95% CI	*P*	OR	95% CI	*P*
Age	1.043	1.010-1.078	0.011	1.066	1.017-1.118	0.008
Gender (male/female)	1.041	0.455-2.381	0.925			
Etiology (hepatitis/other)	0.378	0.165-0.865	0.021			
AFP	1	1.000-1.000	0.898			
HCC (yes/no)	5.417	2.355-12.458	<0.001	7.19	2.077-24.893	0.001
Diabetes (yes/no)	0.846	0.191-3.748	0.826			
History of GIB (yes/no)	1.354	0.546-3.357	0.513			
White blood cell	1.166	1.079-1.259	<0.001	1.123	1.051-1.201	0.001
Platelet	1.002	0.996-1.008	0.502			
Red blood cell	0.26	0.125-0.541	<0.001	0.375	1.131-1.068	0.066
Hemoglobin	0.987	0.968-1.006	0.175			
Albumin	0.902	0.837-0.972	0.007	1.164	1.031-0.913	0.623
Total serum bilirubin	1.013	1.005-1.020	0.001	1.014	1.005-1.023	0.003
Serum creatinine	1.017	1.007-1.026	<0.001	1.012	1.004-1.021	0.006
International normalized ratio	1.009	0.962-1.059	0.712			
Alanine aminotransferase	1.008	1.003-1.012	0.001	1.005	1.000-1.009	0.036
Aspartate aminotransferase	1.004	1.002-1.006	<0.001			
Alkaline phosphatase	1.002	1.000-1.004	0.036	0.794	0.995-1.006	0.794
Gamma-glutamyl transpeptidase	1	0.999-1.002	0.524			
Child-Pugh score	2.219	1.695-2.905	<0.001			
MELD score	2.789	1.743-4.462	<0.001			
NLR score	1.046	1.017-1.075	0.002			
CAGIB score	3.408	2.214-5.244	<0.001			

HCC: hepatocellular carcinoma; OR: odds ratio; CI: confidence interval; AFP: α-fetoprotein; GIB: acute gastrointestinal bleeding; ALT: alanine aminotransferase; AST: aspartate aminotransferase. Child-Pugh score, MELD score, and NLR score are complex variables composed of many clinically significant variables, and therefore, they were not included in the multivariate analysis. ALT and AST had a potential collinearity for assessing liver function, and therefore, AST was excluded in the multivariate analysis.

## Data Availability

The data of this study would be available on request through the corresponding author. The corresponding author is Shuixiang He with the E-mail address hesx123@126.com. The contacted address is 277 West Yanta Road, Xi'an, Shaanxi 710061, China. Fax: +86-85323112. Tel: +86-85323112.
